# Aerosol influence on energy balance of the middle atmosphere of Jupiter

**DOI:** 10.1038/ncomms10231

**Published:** 2015-12-22

**Authors:** Xi Zhang, Robert A. West, Patrick G. J. Irwin, Conor A. Nixon, Yuk L. Yung

**Affiliations:** 1 Department of Earth and Planetary Sciences, University of California Santa Cruz, Santa Cruz, California 95064, USA; 2 Jet Propulsion Laboratory, California Institute of Technology, 4800 Oak Grove Drive, Pasadena, California 91109, USA; 3 Atmospheric, Oceanic and Planetary Physics, University of Oxford, Clarendon Laboratory, Parks Road, Oxford OX1 3PU, UK; 4 NASA Goddard Space Flight Center, Greenbelt, Maryland 20771, USA; 5 Division of Geological and Planetary Sciences, California Institute of Technology, Pasadena, California 91125, USA

## Abstract

Aerosols are ubiquitous in planetary atmospheres in the Solar System. However, radiative forcing on Jupiter has traditionally been attributed to solar heating and infrared cooling of gaseous constituents only, while the significance of aerosol radiative effects has been a long-standing controversy. Here we show, based on observations from the NASA spacecraft Voyager and Cassini, that gases alone cannot maintain the global energy balance in the middle atmosphere of Jupiter. Instead, a thick aerosol layer consisting of fluffy, fractal aggregate particles produced by photochemistry and auroral chemistry dominates the stratospheric radiative heating at middle and high latitudes, exceeding the local gas heating rate by a factor of 5–10. On a global average, aerosol heating is comparable to the gas contribution and aerosol cooling is more important than previously thought. We argue that fractal aggregate particles may also have a significant role in controlling the atmospheric radiative energy balance on other planets, as on Jupiter.

## Abstract (web summary)

Aerosols play an important role in Earth's radiative balance, but their influence on the climate of giant planets is unclear. Here, the authors show that gases alone cannot maintain the energy balance in the middle atmosphere of Jupiter, instead proposing that an aerosol layer dominates radiative heating.

## Graphical abstract

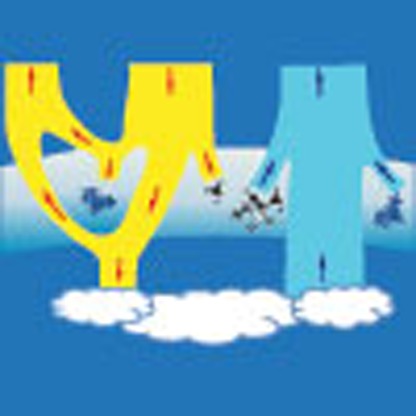

As on Earth, Jupiter's atmospheric temperature profile exhibits a strong inversion above the tropopause^1^, implying that its middle atmosphere, or the ‘stratosphere', is convectively inhibited. Therefore, the energy budget should be dominated by radiation and the stratified middle atmosphere is in global radiative equilibrium. A first-order question is: which constituents in the atmosphere control this energy balance? About half of the incoming solar radiation on Jupiter penetrates deep into the troposphere and one third is reflected back to space (Fig. 1)^2^. The bulk constituents, hydrogen and helium, are not radiatively active except via H_2_–H_2_ and H_2_–He collisional-induced absorption (CIA) at pressures >10 hPa (refs 3, 4). The next most abundant gas, methane (CH_4_), diffuses upward from the deep atmosphere and heats the stratosphere by absorbing the near-infrared solar flux^3^,^4^,^5^,^6^,^7^,^8^. The methane photochemical products acetylene (C_2_H_2_) and ethane (C_2_H_6_), together with H_2_–H_2_ and H_2_–He CIA, absorb the upward mid-infrared radiation from the troposphere and re-radiate it to space, resulting in an efficient net cooling of the middle atmosphere^3^,^4^,^5^,^6^,^7^,^8^,^9^ to compensate the solar heating.

The global maps of temperature and C_2_ hydrocarbons were recently retrieved from the Jupiter flyby data from Cassini and Voyager-1 spacecraft in 2000 (refs 4, 10, 11, 12) and 1979 (refs 4, 12), respectively. On the basis of a state-of-the-art radiative transfer model (see Methods section), we investigate the global energy balance of Jupiter^4^. Surprisingly, the global average cooling flux by gaseous constituents in the middle atmosphere is estimated to be ∼1.4 W m^−2^, about 1.5 times larger than solar flux absorbed by the stratospheric CH_4_ (∼0.9 W m^−2^; Fig. 1). Vertically, the gas solar heating rate is substantially smaller than the gas thermal cooling at pressures >10 hPa (ref. 4). The energy imbalance consistently revealed by the Voyager and Cassini data is not a seasonal effect because Jupiter has nearly zero obliquity. The Jupiter–Sun distance was different for the two flybys, varying from northern fall equinox (Voyager) to the northern summer solstice (Cassini), but the global average heating is not altered significantly. Long-term ground-based observations from 1980 to 2000 also show that the global average temperature at 20 hPa does not substantially vary with time^10^, and thus neither does the thermal radiative cooling. The violation of the radiative energy equilibrium thereby suggests the presence of an additional strong heat source other than CH_4_ in the middle atmosphere of Jupiter, which absorbs the missing ∼0.5 W m^−2^.

Here we show that the missing heat source is aerosols, the end product of atmospheric chemistry on Jupiter. As a result of photochemistry, with the help of auroral chemistry, especially at high latitudes (or the ‘auroral zone') where high-energy particles penetrate into the atmosphere, complex hydrocarbon compounds can form and eventually coagulate and condense as aerosols, or haze particles^13^,^14^. On the basis of Cassini imaging science subsystem (ISS) observations^15^, here we derive the globally averaged solar flux absorbed by the stratospheric aerosols of ∼0.5 to 0.7 W m^−2^, more than half of the amount due to CH_4_ (Fig. 1). The aerosol heating is predominant at middle and high latitudes, exceeding the local gas heating rate by a factor of 5–10. For the first time, we estimate the possible aerosol cooling effect, which might be as important as the cooling via hydrocarbons at high latitudes. We conclude that the aerosols maintain the atmospheric energy balance and must be partially responsible for the stratospheric temperature inversion. That the photochemistry and auroral chemistry control the atmospheric energetics via the production of aerosols suggests that Jupiter exhibits a new regime of atmospheric energy balance that is different from that of the Earth.

## Results

### Aerosol heating effect

The global aerosol map has been revealed from images acquired by the ISS during its Jupiter flyby^15^. At low latitudes (40° S to 25° N), an optically thin layer composed of compacted particles with radii ∼0.2–0.5 μm is found to be concentrated at ∼50 hPa. The rest of the atmosphere is covered by an optically thick aerosol layer at 10–20 hPa composed of fluffy, fractal particles aggregated from hundreds to thousands of ten-nanometre size monomers, similar to the haze particles on Titan^16^,^17^. The fractal dimension of these aggregates is assumed as 2, meaning that their geometric structure of the aggregate particles lies between a long linear chain (fractal dimension of 1) and a fully compacted cluster (fractal dimension of 3). This type of fractal aggregates is consistent with the ISS observations and the polarization observations of Jupiter, whereas spherical particles are not^16^,^17^. Fractal aggregates are known to be much more absorbing than spherical particles in the ultraviolet and visible wavelengths^18^. For instance, from 0.2 to 1 μm, an aggregate particle composed of a thousand ten-nanometre monomers can absorb twice as much of the solar flux as an ensemble of 0.07 μm individual spherical particles (assumed in ref. 19) with the same extinction optical depth, because the former is less scattering than the latter. Figure 2b shows that, at high latitudes, the opacity of the fractal aggregates on Jupiter is considerably larger than the CH_4_ opacity in the ultraviolet and visible ranges, indicating that these particles can absorb a significant fraction of solar energy at wavelengths where the solar blackbody radiation peaks (Fig. 2a). In addition, long-term observations suggest that the seasonal variation of the Jovian north–south polarization asymmetry is only ∼0.5% (ref. 20). The lack of strong temporal variation implies that the fractal aggregates constitute a steady heat source.

The radiative heating calculations demonstrate that the middle atmosphere of Jupiter is heated by two components: aerosols in the ultraviolet and visible wavelengths and CH_4_ in the near-infrared. Figure 2c shows the spectrally resolved zonally averaged heating rate as a function of wavelength and pressure at 60° S. At near-infrared wavelengths longer than 0.9 μm, strong CH_4_ bands completely dominate the heating with minor contributions from H_2_–H_2_ and H_2_–He CIA (Fig. 2b). At shorter wavelengths, heating by the fractal aggregates is predominant. The maximum aerosol heating occurs near the wavelength of the solar spectrum peak (∼0.5 μm), but slightly shortwards owing to the increasing absorption of aerosols towards the ultraviolet. At 60° S, the integrated aerosol heating rate over all wavelengths can reach ∼0.2 K per day at 10 hPa, where it exceeds the CH_4_ heating rate by a factor of 10 (Fig. 3a). The aerosol heating rate appears to decrease rapidly above 5 hPa and does not contribute significantly to the total heating rate in the upper stratosphere. However, this conclusion is not certain since the resolution of the near-infrared spectra^21^ is not sufficient to fully characterize the upper stratosphere. On the basis of higher-resolution observations^22^, another aerosol layer was found above 5 hPa at the poles that might contribute to the local heating rate in the upper stratosphere, though not to the total energy budget of the middle atmosphere due to its lower density at those levels.

**Figure 1:**
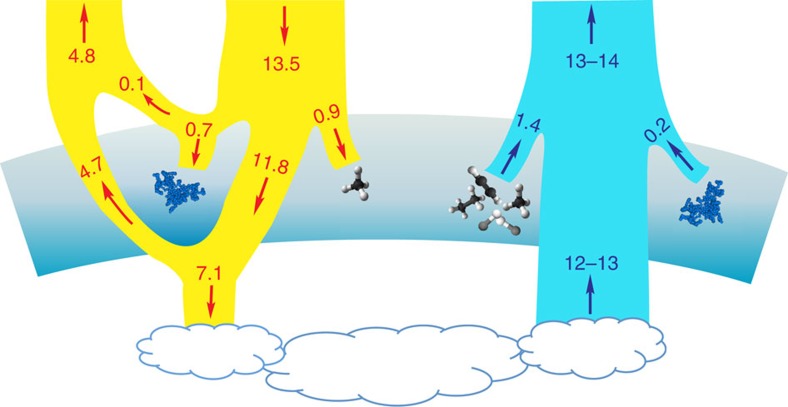
Globally averaged heating and cooling fluxes on Jupiter. The heating (yellow branch) and cooling (cyan branch) fluxes are in units of W m^−2^. The stratosphere is shaded. The heating flux is associated with the incoming solar radiation and the cooling flux is related to the outgoing thermal radiation. Of the 13.5 W m^−2^ of solar radiation incident to Jupiter's atmosphere, 0.1 W m^−2^ is reflected back to space and 11.8 W m^−2^ is transmitted to the troposphere. Tropospheric hazes and clouds absorbed 7.1 W m^−2^ and 4.7 W m^−2^ is reflected back to space^2^. The remainder of the solar energy is absorbed in the middle atmosphere by fractal haze particles (0.7 W m^−2^) and CH_4_ gas molecules (0.9 W m^−2^). The total outgoing thermal radiation from our radiative calculation is ∼13–14 W m^−2^, consistent with that from Cassini and Voyager observations^9^. The thermal cooling flux is mainly emitted from the troposphere (12–13 W m^−2^). In the middle atmosphere, the net cooling flux is 1.4 W m^−2^ emitted by gas molecules H_2_, CH_4_, C_2_H_2_, and C_2_H_6_ (black and white molecule diagrams). The upper limit of the outgoing thermal flux from the fractal aggregates (blue diagrams) is ∼0.2 W m^−2^ as determined in this study.

On the basis of Cassini observations, the globally averaged heat flux absorbed by the stratospheric aerosols is ∼0.5–0.7 W m^−2^, more than half of the amount due to CH_4_ (Fig. 1). The maximum globally averaged aerosol heating rate of ∼0.03 K per day occurs at ∼10 hPa, comparable to the total near-infrared CH_4_ heating rate at the same pressure level. But the globally averaged heating rate including aerosols is about twice that of the gas-only heating rate at pressures >20 hPa (Fig. 3b). Spatially, the aerosol heating is predominant at middle and high latitudes, which is attributable to the optically thick fractal aggregates layer (Fig. 4b). Therefore, the aerosol heating naturally, if not coincidentally, compensates for the energy deficit due to gas heating and cooling at the appropriate pressure levels (Fig. 4e).

Owing to the existence of degenerate solutions in the interpretation of ISS observations^15^, we performed sensitivity tests to estimate the uncertainty ranges of the aerosol heating rate. The tropospheric haze and cloud are treated as an effective cloud layer in the troposphere^15^. Our tests show that the effective cloud albedo and phase function within the retrieved uncertainties has insignificant effect on the stratospheric heating rate. The heating rate is more sensitive to the total optical depth and single scattering albedo of the stratospheric aerosols. Given the constraints from the Cassini ISS observations, testing the sensitivity of the heating rate to each individual aerosol parameter is inappropriate. However, multiple solutions still exist in the aerosol retrieval^15^. Therefore, we adopted five typical retrieval solutions for the middle and high latitudes, namely cases H1–H5 in Table 1. Those cases were designed to explore the parameter space within the uncertainties of the CH_4_ mixing ratio^4^,^23^ and the imaginary part of the refractive indices (hereafter *k* values) of the UV1 and CB3/MIT3 channels of Cassini ISS^15^. All cases provide good fits to the limb-darkening observations from Cassini ISS (Table 1).

The heating rate calculation at 60° S (Fig. 3a) shows that the maximum heating rate including aerosols is about a factor of 2 larger than the minimum at ∼10 hPa. The maximum (H3) and minimum (H2) occurs when the *k* value of the UV1 channel reaches the upper and lower bound, respectively, outside which the UV1 limb-darkening profiles cannot be explained^15^. On a global average (Fig. 3b), the maximum heating rate including aerosols is about a factor of 1.5 larger than the minimum (shaded in red in Fig. 4e,f).

### Aerosol cooling effect

**Figure 2:**
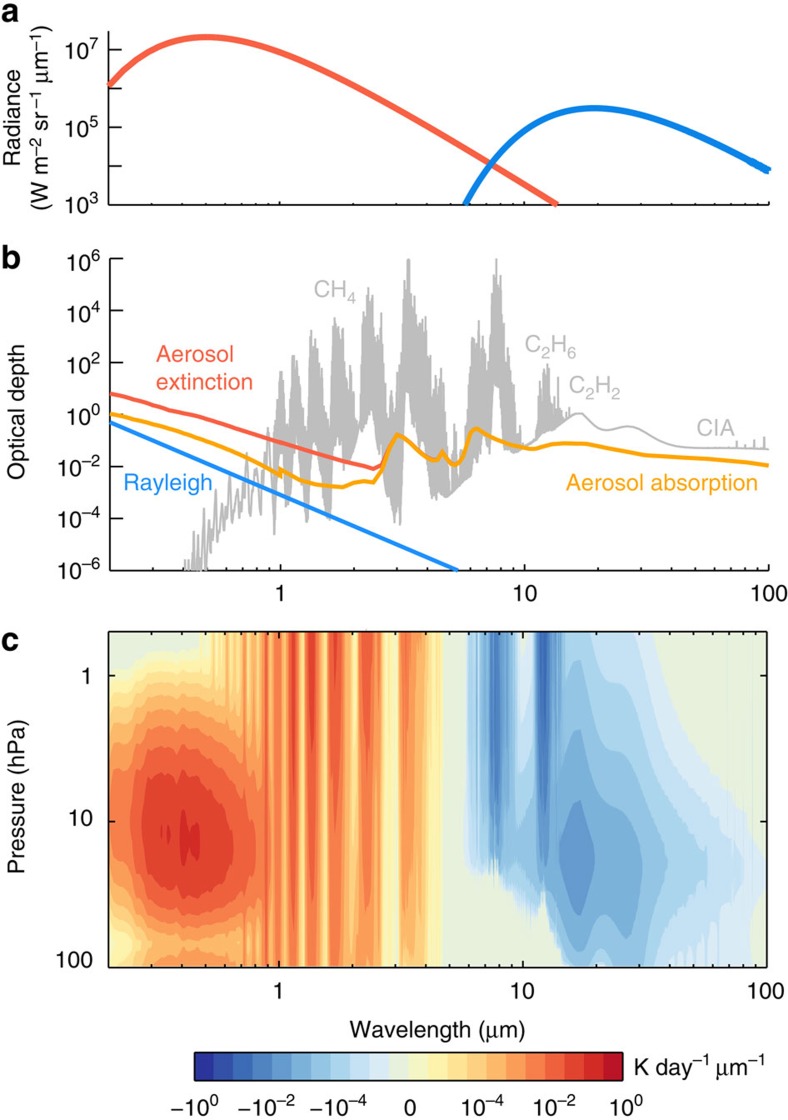
Spectrally resolved heating and cooling rates and corresponding energy fluxes and opacity. (**a**) Globally averaged solar radiation received by Jupiter, approximated by a blackbody of 5,778 K (red) and Jupiter thermal radiation in the stratosphere approximated by a blackbody of 150 K (blue). (**b**) Total optical depth from the top of the atmosphere to 100 hPa as a function of wavelength at 60° S. The gas optical depth (grey) includes H_2_–H_2_ and H_2_–He CIA and CH_4_, C_2_H_2_ and C_2_H_6_ absorption. The non-gas components include Rayleigh scattering (blue), fractal aggregate aerosol extinction (red) and the aerosol absorption (orange). (**c**) Spectrally resolved zonally averaged solar heating (0.2–5 μm) and cooling (5–100 μm) map at 60° S. Absolute values of the heating/cooling rates that are <10^−6^ K per day per μm are not shown. Solar heating dominates shortwards of 5 μm while Jupiter thermal cooling (shown in negative values here) dominates longwards of 5 μm. Contributions from the H_2_–H_2_ and H_2_–He CIA and gas vibrational–rotational bands are shown. Aerosol heating is important in the ultraviolet and visible regions and aerosol cooling is important in the mid-infrared region beyond 11 μm.

**Figure 3:**
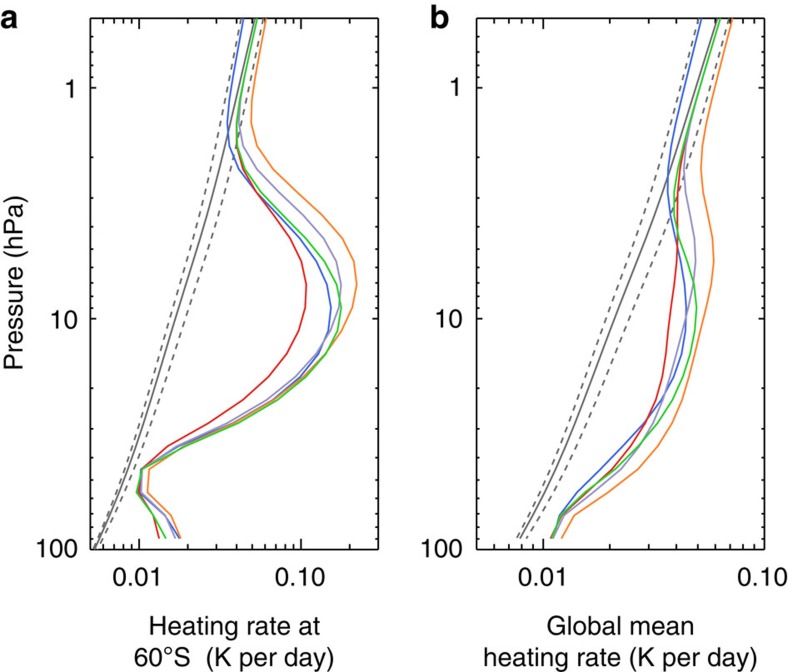
Vertical heating rate profiles. (**a**) Zonally averaged heating rates at 60° S; (**b**) Globally averaged heating rates. The gas-only calculations are shown in black. The dashed black lines show the possible gas heating rates due to the uncertainty of CH_4_ profiles. The coloured lines represent different aerosol retrieval solutions. Cases H1–H5 correspond to the green, red, purple, blue and orange curves, respectively. See Table 1 for detailed input information of the cases.

Aerosols could also cool the middle atmosphere of Jupiter but this effect has not been explored in previous studies. The mid-infrared optical properties of aerosols produced in a hydrogen-dominated environment have not been measured experimentally. But at wavelengths shorter than 2.5 μm, previous laboratory experiments found that the *k* values of aerosols produced in a CH_4_/H_2_ gas mixture could be either larger or smaller than their counterpart in the CH_4_/N_2_ mixture, depending on the chemical composition and environmental pressure^24^,^25^. Therefore, the fractal aggregates on Jupiter might have non-negligible opacity in the mid-infrared compared with H_2_–H_2_ and H_2_–He CIA if their optical constants behave like the aerosols on Titan, which are strongly absorbing with almost no scattering beyond 5 μm (Fig. 2b)^26^,^27^. To estimate the possible cooling effect from aerosols, we included aerosol absorption in a non-linear inversion model to fit the spectra from Cassini CIRS^28^. However, owing to insufficient sensitivity of CIRS observations to the Jovian aerosol opacity, a pure-gas (that is, non-detection of aerosols) model is also able to fit the spectra^4^,^10^,^11^,^12^. Future analysis on the possible C–H bending vibrational features of aerosols at 1,380 and 1,460 cm^−1^ that have been detected on Titan^27^ might provide more constraints on the infrared opacity and chemical structure of aerosol particles on Jupiter. Here we aim to derive the upper limit of aerosol opacity from the CIRS spectra and estimate the upper bound of the aerosol thermal infrared cooling.

For each latitude, we included aerosols in the Non-linear optimal Estimator for MultivariatE spectral analySIS (NEMESIS) model^28^ and retrieved the temperature profile and the mixing ratios of C_2_H_2_ and C_2_H_6_ following the procedure detailed in ref. 4. Owing to the lack of Jupiter-analogue aerosol measurements in mid-infrared, we tested several *k* values based on the laboratory tholin results^26^ and recently derived *k* values from Cassini observations on Titan^27^. The latter shows cooling 2–3 times smaller than the former and exhibits different wavelength dependence. We gradually increased the *k* values until the CIRS spectra cannot be fitted within the measurement uncertainties. Table 2 summarized five typical tests. For each choice of optical constants, we performed an atmospheric retrieval of the Cassini CIRS spectra using NEMESIS model to constrain the aerosol opacity. Figure 5 illustrates the retrieval fitting results and residual values at 57° S and the values of goodness of fit are shown in Table 2. The retrieved temperature profiles are shown in Fig. 6a. If we enhance the optical constants from ref. 25 by a factor of 5 (case C5), we are not able to fit the CH_4_ emission spectra (Fig. 5). Overall, we estimated the upper limit of the aerosol optical depth in the mid-infrared wavelengths to be ∼0.1 at 100 hPa at high latitudes. This places an upper limit on the aerosol contribution to the globally averaged cooling flux of ∼0.2 W m^−2^ in the stratosphere (Fig. 1).

**Figure 4:**
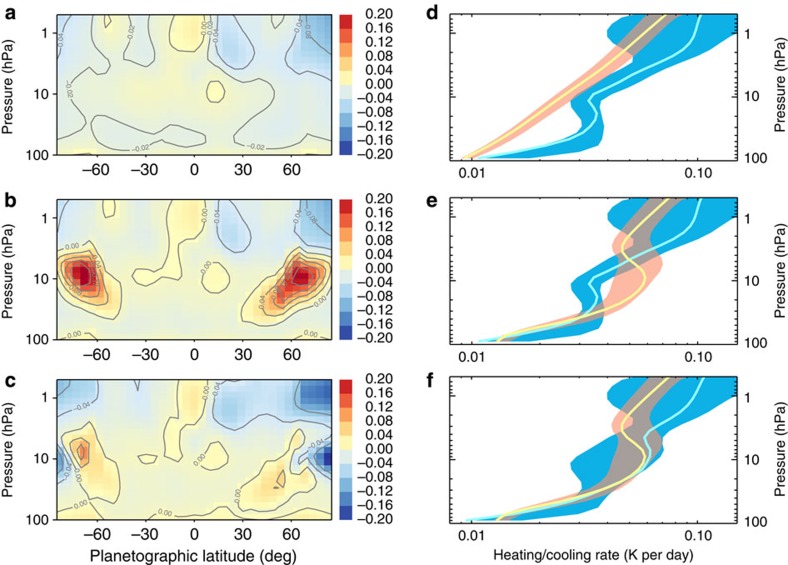
Radiative balance calculation results in the middle atmosphere of Jupiter based on Cassini flyby observations. (**a**) Net radiative heating rate map (in units of K per Earth day) without aerosols; (**b**) net radiative heating rate map with aerosol heating; (**c**) net radiative heating rate map with aerosol heating and cooling; (**d**) globally averaged heating (yellow with pink shading) and cooling (cyan with blue shading) profiles without aerosols; (**e**) globally averaged heating and cooling profiles with aerosol heating; and (**f**) globally averaged heating and cooling profiles with aerosol heating and cooling. The uncertainty ranges are shaded.

**Figure 5:**
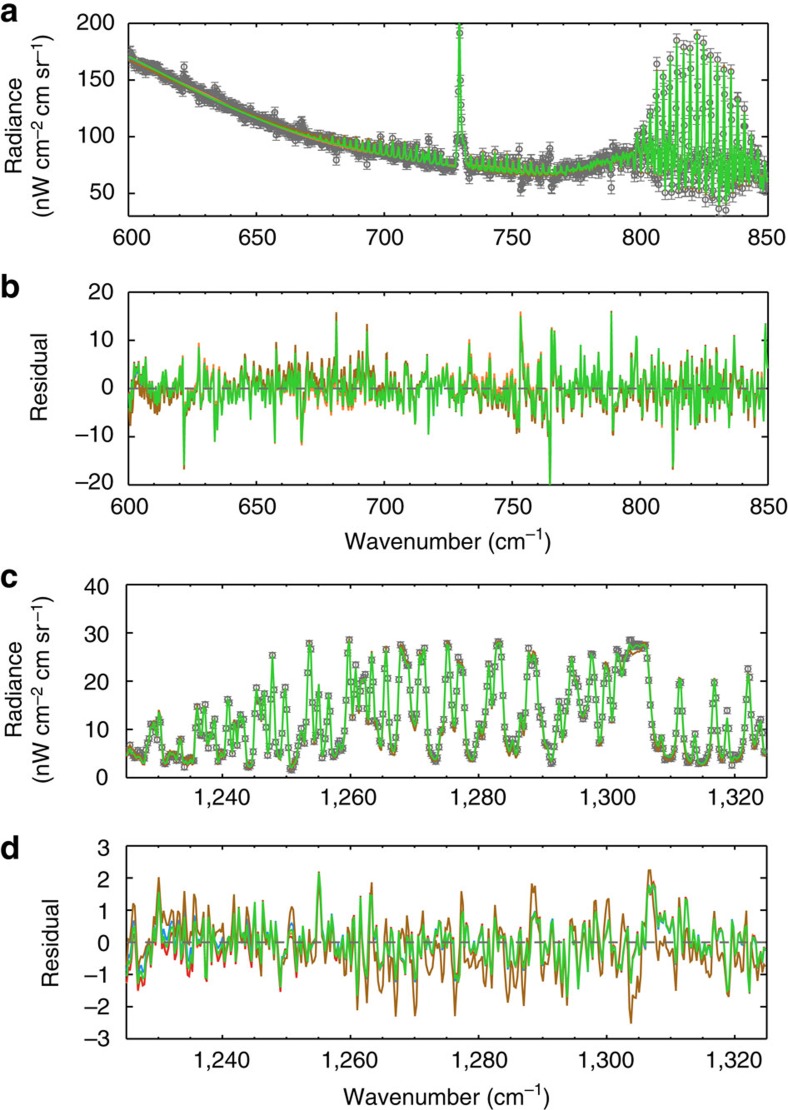
Spectral inversion results at 57° S. (**a**) Spectra at 600–850 cm^−1^ region (H_2_–H_2_ and H_2_–He CIA, C_2_H_2_ and C_2_H_6_ bands); (**b**) fitting residual at 600–850 cm^−1^ region; (**c**) spectra at 1,225–1,325 cm^−1^ region (CH_4_ bands); (**d**) fitting residual at 1,225–1,325 cm^−1^ region. CIRS observations are shown as black circles. The red, blue, orange, green and brown colours represent NEMESIS retrieval cases C1–C5, respectively. The goodness of fit (*χ*^2^/*N* where *N* is the number of measurements) in the 600–850 cm^−1^ region is ∼0.5–0.6 for each case. In the CH_4_ band, the goodness of fit is around unity for each case except for the brown case (*χ*^2^/*N*=1.96), which does not fit the CIRS spectra. See Table 2 for detailed information of the cases.

**Figure 6:**
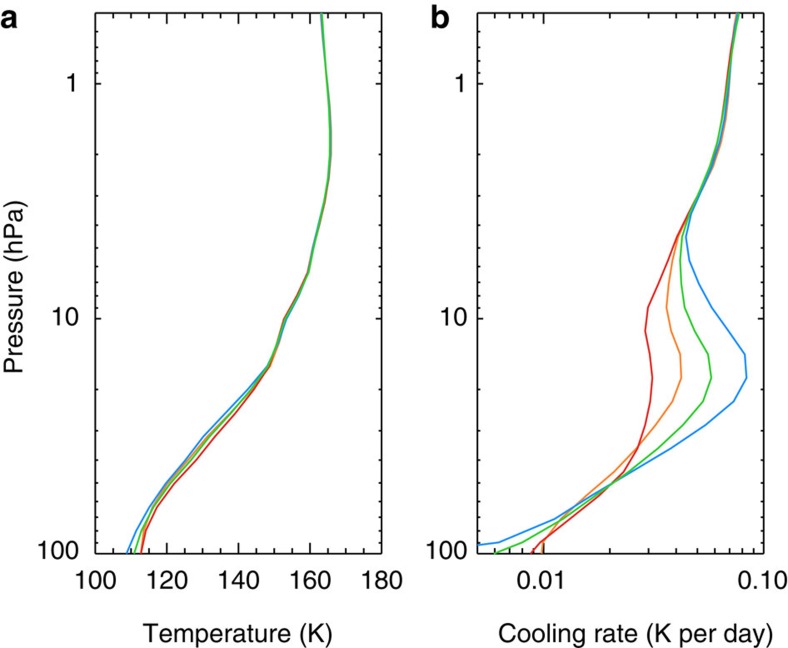
Vertical temperature and cooling rate profiles at 57° S. (**a**) Retrieved temperature profiles; (**b**) corresponding zonally averaged cooling rates. The red, blue, orange and green colours represent cases C1–C4, respectively. The C5 case is not used because it cannot explain the CIRS observations.

The aerosol cooling effect could be significant at middle and high latitudes where the particles are abundant but negligible at low latitudes. When the aerosol opacity is included, the cooling rate increases in the aerosol layer (Fig. 6b). At 57° S, with a moderate choice of *k* (case C4), the zonally averaged cooling rate including aerosols is ∼0.06 K per day. This is about two times larger than the gas-only cooling rate (case C1) at the pressures where the aerosol mixing ratio peaks (∼10–20 hPa, Fig. 6b). The globally averaged aerosol cooling rate could be comparable to the gas cooling rate at 20 hPa and partially compensate for the aerosol heating effect (Fig. 4f). On the other hand, the cooling rates from the aerosol cases are smaller than the pure-gas case at pressure levels below the aerosol layer (Fig. 6b), a result of energy conservation as constrained by the total emission observed by CIRS. Indeed, stronger aerosol absorption leads to a colder retrieved temperature profile (Fig. 6a). For instance, the temperature profile at 57° S from the C2 case is ∼3 K colder than the case without aerosols (C1 case) below ∼40 hPa. This colder temperature leads to a smaller cooling effect than the pure-gas case (C1) at pressures >∼40 hPa where the cooling is dominated by H_2_–H_2_ and H_2_–He CIA (Fig. 6b).

We estimate the possible aerosol cooling rate for each latitude and their influences on the globally averaged cooling rate by combining the ‘ensemble uncertainty'^4^ of the temperature and gas abundances and the aerosol cooling rate tests. The uncertainty range including the aerosol contribution (Fig. 4f) is larger than that without aerosols (Fig. 4d) because the aerosols remain elusive from the CIRS spectra. Global radiative equilibrium is achievable when both aerosol heating and cooling are included (Fig. 4f).

## Discussion

Owing to the lack of sufficient observational evidence before, the importance of aerosol heating in the middle atmosphere of Jupiter has long been a controversial question. It was suggested since the 1970s that the Jovian aerosols might absorb solar radiation and heat the atmosphere^6^,^7^,^8^. In the 1990s, based on the International Ultraviolet Explorer and Voyager-2 data, aerosol heating was shown to have a large impact on the atmospheric circulation^29^. However, a later analysis using Hubble Space Telescope (HST) images found that the aerosols have relatively insignificant effects^19^. With better constraints on the spatial distribution and optical properties of aerosols, Cassini observations confirm a pronounced aerosol heating at high latitudes on Jupiter. Aside from the aerosol cooling effect, our conclusion is qualitatively consistent with previous estimate from the International Ultraviolet Explorer and Voyager-2 data^29^ but with a much better spatial coverage. However, this work disagrees with heating rates derived solely on the basis of HST data^3^,^19^.

The major difference between the current work and previous studies is probably attributable to the fractal nature of the aerosol and aerosol spatial distribution. Through the multi-channel–multi-phase retrieval on Cassini images, we can characterize the fractal aggregates in great detail, including the optical depth, single scattering albedo and phase function of the particles. On the basis of low-phase-angle images alone^19^, the polar aerosols were assumed as tiny spherical particles of ∼0.07 μm in radius instead of the submicron size aggregate particles. The latter have lower single scattering albedo than the spherical particles. Furthermore, with a larger particle size, the fractal aggregates have less backscattering than the spherical particles. Owing to the above two reasons, a larger total optical depth of the fractal aggregates is required to explain the low-phase-angle I/F observations, leading to a larger heating rate in this study, than that in ref. 19, at the south pole. Note that the *k* values used in ref. 19 are lower than our nominal model but still within the sensitivity test range in our study.

The vertical distributions of aerosols in previous studies^19^,^29^ are based on microphysical simulations that are inconsistent with the near-infrared observations^21^. Previous studies adopted a haze layer located above 10 hPa at polar regions, while the near-infrared spectra reveal a main haze layer at 10–20 hPa (ref. 20). West *et al*.^29^ only sampled two latitudes and estimated the other latitude information by scaling. The HST images^19^ have a good latitudinal coverage, but the aerosol heating appears influential only at the south pole, not at middle latitudes or at the north pole. Moreno *et al*.^19^ reported the aerosol optical depth at the north pole one order of magnitude less than that at the south pole. This result is inconsistent with other observations. For example, recent high-resolution ground-based near-infrared spectra^22^ concluded that the near-infrared haze optical depth at the northern pole is comparable to that at the southern pole. The Cassini images in low and high phase angles also revealed that the haze ultraviolet optical depth at north high latitudes is not significantly less than its south counterpart^15^. Furthermore, Cassini images show that the haze optical depth in the ultraviolet channel can approach unity down to 100 hPa at middle latitudes^15^. Including the haze contribution at those latitudes would greatly enhance the aerosol heating. This will not only influence the local heat balance but also on the global energy equilibrium, especially at 10–20 hPa, as shown in our study.

Several other factors might also attribute to the difference between the current work and previous studies. For instance, we have a much better global coverage of the temperature, hydrocarbons and aerosols based on Cassini observations. The cooling rate in ref. 3 was likely to be underestimated because the temperature profile from Galileo entry probe is shown to be colder than the globally averaged temperature profile from Voyager and Cassini observations^4^, albeit the cooling rate in ref. 3 is still slightly larger than the gas heating rate at pressures >10 hPa. The spectroscopic data of CH_4_, C_2_H_2_ and C_2_H_6_ have been greatly improved in the last decade (see Methods section for gas opacity). The state-of-the-art line data allow us to adopt the line-by-line approach to resolve the vibrational–rotational line shape of hydrocarbons^4^, the most accurate radiative transfer method to estimate the gas heating and cooling rate.

Another possible heating mechanism in the middle atmosphere is energy dissipation of upward propagating gravity waves from the troposphere. However, as per previous studies^30^,^31^, this hypothesis has several defects. First, there is little evidence of stratospheric gravity waves at middle and high latitudes. Second, there is no direct evidence of wave breaking in the lower stratosphere of Jupiter. Third, gravity wave breaking could either heat or cool the middle atmosphere but the net effect is difficult to quantify^30^.

Unlike the Earth, on which the photochemical product (ozone) only dominates the atmospheric radiative heating, Jupiter might exhibit a different regime of atmospheric energy balance where both the heating and cooling are significantly controlled by the photochemistry and auroral chemistry via the production of aerosols and C_2_ hydrocarbons. Aside from the first-order global energy balance, aerosol heating and cooling on Jupiter also influence the spatial distributions of radiative forcing, which has a significant impact on the large-scale dynamical circulation in the middle atmosphere^5^,^19^,^29^. The NASA JUNO spacecraft, arriving at Jupiter in 2016, will provide more insights on the aurora processes and aerosol formation in the polar region.

Jupiter is the second planetary body and the first hydrogen-dominated planet that shows evidence of hydrocarbon aerosols playing a significant role in regulating the radiation flux and most probably the circulation of its middle atmosphere. The other one is Titan^32^. Although Jupiter's atmosphere is primarily dominated by hydrogen, Titan's is dominated by nitrogen, both of these atmospheres produce fluffy, fractal aggregate particles, suggesting that fractal aggregates might be a ubiquitous result of hydrocarbon chemistry. In view of the existence of hydrocarbon aerosols in many other atmospheres dominated by hydrogen or nitrogen, such as those of Saturn^33^, Uranus^34^ and Neptune^35^, the early Earth^18^, and possibly some exoplanets^36^,^37^,^38^,^39^,^40^, we hypothesize that the heating and cooling from fractal aggregates could also be important for determining the radiative energy distribution and climate evolution on these planets. Owing to their strong heating effects, fractal aggregates play a significant role in creating the temperature inversion in the lower stratosphere of Jupiter. They might also be partially responsible for the temperature inversions observed on the other giant planets in the Solar System, but neglected in previous studies^41^. A typical feature of the fluffy aggregate particle is its extremely low density, which might help to explain the existence of very high and thick haze layers at pressures <0.1 hPa, as seen on the Neptune and sub-Neptune size planets GJ436b (ref. 39) and GJ1214b (ref. 40). A thorough study of fractal aggregates will shed light on how to characterize these particles in future photometry and polarization observations.

## Methods

### Radiative heating and cooling model

For heating rate calculations between 0.20 and 0.94 μm where the aerosol contribution is significant, we use a multiple scattering model based on the C version of the discrete ordinates radiative transfer code (DISORT Program for a multi-layered plane-parallel medium)^42^. The phase function and cross sections of low-latitude particles were calculated based on Mie theory, while that of the fractal aggregates at middle and high latitudes were computed using a parameterization method for the aggregates with a fractal dimension of two^17^. The parameterization is based on electromagnetic scattering computations using the multi-sphere method^43^. In the heating rate calculations, we use 32 streams to characterize the intensity angular distribution, which displays almost no difference from the 64-stream case. A Gaussian quadrature method with 10 zenith angles is used to average the heating rates longitudinally. The spectral resolution is 0.001 μm. The effective cloud albedo in the troposphere is interpolated between 0.20 and 0.94 μm based on the retrieved albedo from the UV1 channel and CB3/MT3 channels of Cassini ISS^15^.

At longer wavelengths (0.94–200 μm), our calculations adopt the line-by-line approach, based on a state-of-the-art high-resolution radiative heating and cooling model for the stratosphere of Jupiter, which has been rigorously validated against simple but realistic analytical solutions^4^. The CH_4_ heating rate calculation from 0.94 to 10 μm is performed with a spectral resolution of 0.005 cm^−1^ to resolve the CH_4_ spectral line shape using the most current CH_4_ line lists (see discussion for gas opacity below). The thermal cooling rate from 50 to 2,500 cm^−1^ (4–200 μm) is calculated with a spectral resolution of 0.001 cm^−1^.

We calculate heating and cooling rates for every latitude to produce the latitude-pressure two-dimensional maps. The specific heat of Jupiter's atmosphere is taken as 1.0998 × 10^4^ J kg^−1^ K^−1^ (ref. 44). The globally averaged profiles are obtained via an area-weighted mean from 90° N to 90° S. Since the data quality at latitudes north of 70° N and south of 70° S is not sufficiently good for rigorous atmospheric retrieval, we do not derive the atmospheric characteristics from the ISS images and CIRS spectra. Instead, we assume that the vertical profiles of aerosol, temperature and gases are identical to their values at 70° north and south, respectively.

This assumption might introduce some uncertainty in our estimate of the global energy balance because the heating and cooling in the polar region, especially in the infrared aurora region, are not negligible. The polar aurora is known to be highly variable both temporally and spatially^1^. According to our limited data on the polar regions from Galileo^45^ and Cassini^46^ spacecraft as well as ground-based observations^47^,^48^, both regions poleward of 70° N and 70° S could be different from that ∼65°–70°. For example, the Cassini CIRS instrument^46^ detected a variation of thermal emission over the C_2_H_2_ and C_2_H_6_ bands at regions poleward of 65°, within about a factor of 2. However, because the surface area poleward of 70° amounts to merely 6% of the total surface area of Jupiter, increasing the polar cooling rate by a factor of 2 will probably introduce an uncertainty of only ∼6% of the total cooling rate, which is still well-located within our estimated uncertainty range (Fig. 4f). On the other hand, the pressure level of infrared aurora source has not been precisely determined. If the emission originates from the upper stratosphere (for example, above 0.1 mbar level), the aurora and its variability might have little impact on the thermal cooling rate at pressures we focus here. We should also point out that the aurora might also be associated with some heating mechanisms in the polar region that have not been considered in this study. Future analysis of the Jovian polar region will provide more information on the local energetics.

The aerosol and gas opacities used in the radiative calculations are discussed below.

### Aerosol opacity sources

In the visible and near-infrared wavelengths, the early laboratory studies^24^,^25^,^26^ measured the optical properties of Titan-analogue aerosols (that is, in the nitrogen environment) and Jupiter-analogue aerosols (that is, in the hydrogen environment), respectively. Using the radio frequency glow discharge in a CH_4_/H_2_ gas mixture, it was found that the refractive indices of these aerosols are consistent with high-phase-angle photometry data of Uranus by Voyager-2 at 0.55 μm (ref. 24). Compared with aerosols produced in the CH_4_/N_2_ mixture^26^, the imaginary part of the refractive index of the Jupiter-analogue aerosols could be either larger or smaller, depending on the chemical composition. Unfortunately, ref. 24 has been the only laboratory experiment of the CH_4_/H_2_ gas mixture to date. It should be noted that the subsequent Titan-analogue laboratory measurements show that the aerosol properties are significantly influenced by the gas composition and environmental pressure^25^.

Zhang *et al*.^15^ combined the ground-based infrared spectra with the Cassini ISS observations at both low and high phase angles, and determined the *k* values for the UV1 channel (0.258 μm) and the infrared channels (CB3 at 0.938 μm and MT3 at 0.889 μm). Owing to the existence of degeneracy, the *k* value at UV1 channel varies from 0.008 to 0.02 and that at 0.9 μm varies from 0.0001 to 0.004. Different choices of *k* would imply different solutions to fit the ISS data, such as the radius of the monomers from 10 to 40 nm, the number of monomers per aggregate particle from 100 to 1,000, and the abundance of particles. In all possible solutions, the total aerosol opacity only changes by ∼30% among all solutions. The corresponding aerosol heating rate does not change significantly.

**Figure 7:**
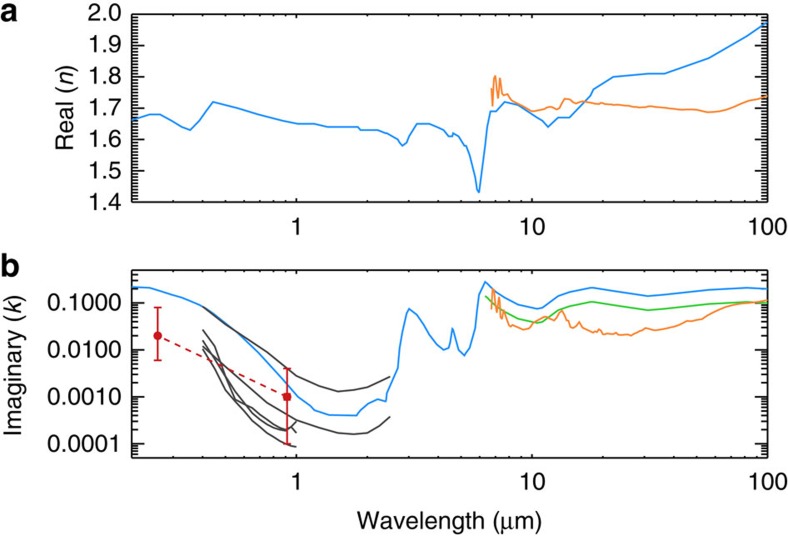
Refractive indices of aerosols from 0.2 to 100 μm. (**a**) Real part of the refractive index; (**b**) imaginary part. The blue one is from the Titan tholin experiment^26^; orange is from Titan CIRS observations^27^; green is same as the blue one but reduced by a factor of 2 (case C4); black is the Jupiter-analogue aerosol experiment^24^; red is the derived from Cassini ISS observations^15^, with an interpolation in the coordinate of linear wavelength and logarithmic *k* value. The red dashed line is used in the nominal case for the heating rate calculation (case H1) in this study.

For the heating rate calculations, we choose the model parameters for the *k* values from ref. 15 as our nominal case (H1 in Table 1), with a careful sensitivity study in the radiatiave heating calculation. The *k* value is ∼0.02 at 0.258 μm and 0.001 at ∼0.9 μm. We performed an interpolation for the wavelengths between 0.20 and 0.94 μm in the coordinate of linear wavelength and logarithmic *k* value. The interpolation can be justified by the approximate same trend shown in the laboratory measurements (Fig. 7). As in ref. 15, we adopt the real part of refractive index (*n*) from ref. 26.

### Gas opacity sources

From 0.20 to 0.94 μm, the CH_4_ opacity is based on ref. 49 and Rayleigh scattering optical depth is taken from ref. 14. From 0.94 to 10 μm, a line-by-line calculation is performed. We compared several CH_4_ line databases, including the HITRAN2012 (refs 50, 51), the database from ref. 22, and the M5 database^52^. For the CH_4_ broadening width, it has been suggested that line widths in the Jovian atmosphere (H_2_–He mixture) are similar to those in the Earth's atmosphere (N_2_–O_2_ mixture)^22^. All the above three CH_4_ opacity sources result in a consistent heating rate in our calculation. However, none of the above opacity sources covers the CH_4_ band between 0.94 and 1.1 μm. Recently, a ‘10 to 10' CH_4_ line database is computed from first principles^53^. This database is shown to be roughly consistent with the HITRAN2012 CH_4_ data in their overlapping near-infrared wavelengths (personal communication with J. Bailey) and therefore is helpful to fill the gap between 0.94 and 1.1 μm in our calculation. For this CH_4_ band, due to the lack of laboratory measurements of line shape parameters, we adopted an average pressure-broadened half-width of 0.06 cm^−1^ for foreign-broadening and 0.077 cm^−1^ for self-broadening at 1 bar pressure, and the temperature dependence exponent is ∼0.85 (refs 52, 54). The contribution from this band to the total heating rate is negligible (Fig. 2c). For the heating rate calculation, we obtain the near-infrared H_2_–H_2_ CIA absorption from refs 55, 56 and H_2_–He CIA absorption from refs 57, 58, 59, 60.

The thermal cooling rate is calculated from 50 to 2,500 cm^−1^ (4–200 μm). The opacity sources of CH_4_, C_2_H_2_ and C_2_H_6_ are obtained from HITRAN2012 with hydrogen-broadening widths^4^,^61^. Fractal aggregates are treated as pure absorbers in the thermal wavelengths due to their negligible single scattering albedo in the mid-infrared. The mid-infrared H_2_–H_2_ and H_2_–He CIAs are obtained from ref. 62. Figure 2c shows the spectrally resolved heating/cooling rate as a function of wavelength and pressure, in which one can see the contributions from the CIAs and different vibrational–rotational bands from gases in Fig. 2b.

### Spectral inversion model

The temperature and hydrocarbon distributions are simultaneously retrieved from the Cassini CIRS spectra^4^,^10^,^11^,^12^ using the NEMESIS algorithm^28^. This inversion model has been used in previous studies involving CIRS data retrieval^4^,^11^,^12^. In this study we extended the previous retrieval framework to include aerosol absorption in the mid-infrared wavelengths. No scattering calculations were needed as the fractal aggregate particles are significant absorbers and have negligible single scattering albedos at these wavelengths.

## Additional information

**How to cite this article:** Zhang, X. *et al*. Aerosol influence on energy balance of the middle atmosphere of Jupiter. *Nat. Commun.* 6:10231 doi: 10.1038/ncomms10231 (2015).

## Figures and Tables

**Table 1:** Sensitivity cases for ISS retrieval and heating rate calculation.

**Case**	**CH**_**4**_ **mixing ratio**	***k*** **(UV1)**	***k*** **(CB3/MT3)**	**Colour**
H1 (Nominal)	1.8 × 10^−3^	2 × 10^−2^	1 × 10^−3^	Green
H2	1.8 × 10^−3^	6 × 10^−3^	1 × 10^−4^	Red
H3	1.8 × 10^−3^	8 × 10^−2^	4 × 10^−3^	Purple
H4	1.5 × 10^−3^	2 × 10^−2^	1 × 10^−3^	Blue
H5	2.5 × 10^−3^	2 × 10^−2^	1 × 10^−3^	Orange

**Table 2:** Sensitivity cases for CIRS retrieval and cooling rate calculation.

**Case**	***k*** **values in the mid-infrared**	***χ***^***2***^***/N*** **(600–850 cm**^−**1**^)	***χ***^***2***^***/N*** **(1**,**225–1**,**325 cm**^−**1**^)	**Colour**
C1	Pure gas, no aerosol	0.544	1.094	Red
C2	Titan tholin experiment^26^	0.543	1.042	Blue
C3	Titan CIRS observations^27^	0.541	1.090	Orange
C4 (Nominal)	C2 values divided by 2	0.543	1.051	Green
C5	Five times of C2 values	0.607	1.964	Brown
